# Hormonal Therapies in Multiple Sclerosis: a Review of Clinical Data

**DOI:** 10.1007/s11910-023-01326-7

**Published:** 2023-12-16

**Authors:** Stephanie Hsu, Riley Bove

**Affiliations:** UCSF Weill Institute for Neuroscience, Division of Neuroimmunology and Glial Biology, Department of Neurology, University of California San Francisco, San Francisco, CA USA

**Keywords:** Multiple sclerosis, Exogenous hormone therapy, Menopausal hormone therapy, Contraceptives, Gender affirming therapy, Fertility treatments

## Abstract

**Purpose of Review:**

Given the potential for exogenous hormones to influence risk and course of MS, this narrative review aims to summarize current knowledge from observational and interventional studies of exogenous hormones in humans with MS.

**Recent Findings:**

Large randomized clinical trials for combined oral contraceptives and estriol both show modest effect on inflammatory activity, with the latter showing potential neuroprotective effect. After fertility treatment, large actively treated cohorts have not confirmed any elevated risk of relapse. Preclinical data suggest that androgens, selective estrogen receptor modulators (SERMs), and selective androgen receptor modulators (SARMs) may be neuroprotective but clinical data are lacking. Gender affirming treatment, particularly estrogen in trans-women, could possibly be associated with elevated risk of inflammation. For women with MS entering menopause, hormone therapy appears safe during the appropriate menopausal window, but its long-term effects on neuroprotection are unknown.

**Summary:**

Exogenous hormones, used in varied doses and for diverse indications, have variable effects on MS risk, inflammatory activity, and neuroprotection. Large randomized trials are needed before it is possible to determine the true effect of exogenous hormones in a condition as complex as MS.

## Introduction

Many lines of evidence suggest that hormones, and particularly gonadal hormones, modulate the risk and course of multiple sclerosis (MS). Classical epidemiological observations include a 3:1 female: male sex ratio among individuals with MS onset after the age of puberty and a decreased risk of relapses in women during the immunotolerant state of pregnancy followed by an increased risk of inflammatory activity postpartum. In animal models, estrogens, androgens, and progestogens all appear to have important effects on relevant immune activation or neurological resilience pathways [1]. These observations have led to substantial interest in the potential immunomodulatory or neuroprotective role of exogenous hormones, and many patients ask about the potential safety and/or efficacy of hormonal treatments for a range of indications. To date, reliable clinical and translational guidance is lacking.

The purpose of this narrative review is to summarize current knowledge gleaned from observational and, most critically, interventional studies of exogenous hormones in humans with MS. The clinical indications for hormones evaluated are contraceptives, menopausal hormone therapy, hypoandrogenism, fertility treatments, and gender affirming treatment. However, to accurately understand the effect of exogenous hormones on MS, there are several challenges in evaluating the mechanistic effect of dynamic hormone levels on a neurological disease as complex as MS. These challenges include (1) differences in endocrine regulation between preclinical animal models and humans; (2) lack of ability to quantify levels of estrogens and aromatized androgens in the central nervous system in vivo or the distribution, density, and affinity of various receptors; 3) pleiotropic effects of hormones on individual and interacting cell types (glial cells, neurons, macrophages); and (4) determining treatment doses given marked differences in circulating hormone values between individuals even within similar life ages and stages. Therefore, potential impact of hormones is measured mostly in the cruder categories of: MS symptoms, MS inflammatory activity (clinical relapses, new MRI lesions), and MS progression (brain atrophy, functional decline). Since older studies of exogenous hormones—primarily oral contraceptives, OCs—evaluated doses typically higher than current doses, this review drew primarily from articles presenting primary clinical data in humans published since 2010.

## Hormonal Contraceptives

To provide context to the literature on exogenous hormones and MS, a brief overview of types of exogenous hormones typically used for contraception is provided (reviewed in (Colquitt et al., 2017). Hormonal oral contraceptives (OCs) are the most commonly used form of contraception in the USA, with 99% efficacy. They consist of either synthetic forms of progestin only, or a combination (combined OCs or COCs) of both synthetic estrogens (20–50 ug per tablet) and progestins in varying amounts. Cyclic OCs are given as active ingredient for 21 days, followed by 7 days of placebo, i.e., inert tablets. OCs come in one of three forms: monophasic, with a fixed amount of hormone in every active tablet; biphasic, with 2 different doses of estrogen and progestin; and triphasic, with 3 different doses of estrogen and progestin, created to better mimic the menstrual cycle’s hormonal fluctuations [2]. Continuous OCs provide one same dose of hormones for 3 months, followed by 7 days of placebo (16), thereby potentially lowering the overall fluctuation in hormone levels compared with cyclic OCs or endogenous cycling.

Lower dose formulations have reduced side effect profiles, although about 40% of women still perceive side effects. Cardiovascular events are associated with all hormonal contraceptive use, although lower dose estrogen formulations (under 50 ug) of OCs are associated with reduced risk of venous thromboembolic events and ischemic stroke. Combination OCs are associated with increased risk of breast cancer up to 10 years after discontinuation, but conversely OCs lower risk of ovarian and endometrial cancer with increasing use. Progestin-only contraception is safe for use in lactation immediately in postpartum [2].

As an alternative to OCs, which require daily adherence, the subdermal implant is a small surgically implanted rod containing 68 mg of the progestin etonogestrel, which is slowly released over 3 years (60–70 ug initially to 30 ug). This is considered the most effective contraceptive, with a 0.05% yearly failure rate. Side effects include irregular menstrual bleeding, changes in libido, skin scarring and infection at the site of the implant, and weight gain [2]. Injectable contraceptives come in the form of medroxyprogesterone acetate, a derivative of progesterone; 150 ug is injected every 3 months. Side effects include menstrual irregularity and changes, delay in return to fertility up to 18–24 months, and weight gain [2]. The intravaginal ring is inserted vaginally and left in place for 3 weeks, with a 1 week period of removal before reinsertion for another 3 weeks. An average of 0.120 mg of etonogestrel and 0.015 mg of ethinyl estradiol is released each day that the ring is in place. The most common side effect are headaches, with more uncommon side effects related to libido, vaginal discharge, nausea, and weight gain [2]. The transdermal patch is applied weekly for 3 weeks, with the fourth week without patch use. It is formulated as a combination of synthetic hormones norelgestromin (releasing 150 ug a day) and ethinyl estradiol (releasing 35 ug a day), and side effects are similar to those of combination OCs [2].

Some intrauterine devices (IUDs) contain hormones that are released directly into the uterus; copper IUDs are the nonhormonal exception. Levonorgestrel-releasing IUDs come in two forms: one that contains 13.5 mg of levonorgestrel and is adequate for 3 years of contraception, and the other containing 52 mg adequate for 5 years use. Similar to other progestin-only contraceptives, levonorgestrel-IUDs can be used during breastfeeding immediately in the postpartum period. Common side effects include irregular bleeding, abdominal and pelvic pain, acne, ovarian cysts, and headache, and adhesion to or perforation of the uterine lining is possible [2]. While the effects of hormonal IUDs should theoretically be localized to the uterine area, studies have suggested they have more systemic impact than anticipated, including hormonal stimulation of breast tissue [3, 4].

### Impact on Inflammation

#### Observational Studies

Recent observational studies have suggested that overall, combined oral contraceptives do not appear to have any negative effects on—but also no clear protective effects against—inflammatory activity. A single-center analysis of combined OC use in 162 prospectively followed women newly diagnosed with RRMS and started on either interferon therapy or glatiramer acetate revealed no significant difference in subsequent annualized relapse rates in a 3-way analysis of (1) prior OC users, (2) current OC users, and (3) never users (*p* = 0.057) [5]. In a second single-center analysis in 495 women presenting with clinically isolated syndrome who were prospectively followed, exposure to OC use was not associated with the risk of either a second attack or of disability accrual [6•]. While these studies suggested overall little effect of combined OCs on risk of inflammatory activity, a further question is whether continuous OCs, which as noted above reduce the total amount of menstrual cycles to 4 instead of 12 annually, could by reducing hormonal shifts also stabilize inflammatory activity. In one small retrospective single-center study of MRI inflammatory activity in 46 women using continuous versus cyclic combined oral contraceptives, women on continuous OCs with at least 1 year of follow-up data showed a significant difference in time to T2 lesion formation (*p* = 0.03) and time to contrast-enhancing lesion formation (*p* = 0.02) compared with women on cyclic OCs [7].

#### Interventional Studies

Three interventional studies have been conducted using various formulations in women with MS.

- **Contraceptive-dose hormones.** A multicenter trial of combined oral contraceptives as an add-on to interferon-β-1a SC was conducted in 150 adult women aged 18–45 with relapsing–remitting MS [8]. Participants were randomized in a 1:1:1 ratio to receive IFN-β-1a subcutaneously (SC) with either (1) no OC, (2) low-dose estrogen combined OC (ethinylstradiol 20 μg and desogestrel 150 μg), or (3) high-dose estrogen combined OC (ethinylestradiol 40 μg and desogestrel 125 μg). Patients were treated for 96 weeks. For the primary outcome, the estimated number of cumulative combined unique active lesions at week 96, the high-dose estrogen group experienced a relative reduction of 26.5% (*p* = 0.04) compared with the no-OC group. Differences between the low-OC group and the no-OC group were not significant. The study was not powered to detect an effect on clinical relapses or sustained disability progression [8].
- **Pregnancy-dose hormones.** Two studies were specifically informed by the relative risk reduction of relapses during the immunotolerant state of pregnancy and sought to determine whether hormones associated with this condition, namely, the “pregnancy estrogen” estriol, or progesterone, could lead to reduction in relapses.
  - Estriol. Following promising results from a pilot trial, a Phase II trial of estriol as an add-on to glatiramer acetate in 164 adult women aged 18–50 with relapsing–remitting MS was conducted at 16 academic neurology centres in the USA between June 28, 2007, and Jan 9, 2014 [9•]. Participants were randomized 1:1 to (1) glatiramer 20 mg SC daily with placebo or (2) glatiramer with daily oral estriol (8 mg) for 24 months. Estriol dosing was intended to mimic induce a mid‐pregnancy blood estriol level. The primary endpoint was annualized relapse rate after 24 months, with a significance level of *p* = 0.10. The annualized confirmed relapse rate was 0.25 relapses per year (95% CI 0.17–0.37) in the estriol group versus 0.37 relapses per year (0.25–0.53) in the placebo group (adjusted rate ratio 0.63, 95% CI 0.37–1.05; *p* = 0.077). Additional potential benefits (fatigue) and concerns (irregular menses) were noted [9•]. An analysis of serological data from 111 participants was performed, evaluating serum neurofilament light chain levels (sNFL), which can reflect nerve injury due either to inflammatory activity or to neurodegeneration [10]. There was a significant decrease in sNFL levels at 12 months for the Estriol + GA group (*t*(109) = 4.10, *p* < 0.0001), while the Placebo + GA group did not change significantly (*t*(109) = 0.41, *p* = 0.7). Further, the two treatment groups differed significantly in NfL at 12 months (*t*(109) =  − 2.15, *p* = 0.03), but not at 6 months (*t*(109) = 0.51, *p* = 0.6) [11]. These levels were not adjusted for habitus, and it was not possible to distinguish possible effects of estriol on neuroprotection or neuroinflammation. One important concern not evaluated in the trial was of what would happen to inflammatory activity after discontinuation of the high-dose estriol; in other words, might an inflammatory rebound be noted as is seen after pregnancy [12, 13]?
  - Progesterone**.** A second trial, the Prevention of Post-Partum Relapses with Progestin and Estradiol in Multiple Sclerosis (POPART'MUS) trial [14], sought to reduce specifically the risk of inflammatory activity in the postpartum period by administering progestins as well as estradiol. This was a randomized 12-week placebo-controlled double-blind clinical trial in adult women with RRMS and SPMS, who were treated with high-dose progestin (19-nor-progesterone derivative nomegestrol acetate 10 mg/day), combined with transdermal 17-beta-estradiol (75 μg, once a week), within 24 h of delivery and for 12 weeks. The blind 12-week period was followed by a 12-week open untreated period. The high-dose progestin was intended to lead to a plasma concentration which is fairly similar to that reached during pregnancy. Enrollment was stopped early due to low recruitment. No treatment effect was observed on ARR after 12 weeks (hormone arm = 0.90 (0.58–1.39), placebo arm = 0.97 (0.63–1.50) (*p* = 0.79))). According to the authors, trial limitations included slow recruitment, arbitrary choice of hormonal agents without adequate dose-finding approach, such that the plasma concentration was substantially less than that targeted, as well as a very short follow-up period insufficient to detect treatment effects on the ARR outcome [15].

### Impact on Neuroprotection

#### Interventional Studies

Few studies have evaluated an effect of exogenous hormones on aspects of neuroprotection in premenopausal female patients. Here, the review focused on recent interventional studies.

- **Contraceptive doses.** In a secondary analysis of MRI data from the multicenter randomized controlled trial of OC combined with interferon beta mentioned above [8], at month 24, the proportion of patients with cognitive impairment was lower in the group of patients taking high-dose estrogen combined OC (34.8%) than in the no-OC group (47.6%) (*p* = 0.03). The risk of developing cognitive impairment over 24 months was also lower in the high-estrogen group (*p* = 0.02). Beyond this representing a secondary post hoc analysis, other limitations of the study were that it was not possible to determine whether the cognitive benefits observed were due to decreased inflammatory activity in this group or to effects on neural plasticity and neurogenesis.
- **Estriol.** In a post hoc analysis of the abovementioned trial of estriol as an add-on to glatiramer acetate [11], no difference in change in T2 lesion volumes was noted over time, but a reduction in cerebral cortex atrophy at 12 months was noted in the estriol group compared with the placebo group. Furthermore, patients in the estriol group without enhancing lesions had less cortical gray matter atrophy than did those in the placebo group, suggesting a direct neuroprotective effect independent from anti-inflammatory effects. Taken together with the nFL data, this was interpreted as evidence of a protective effect of estriol from neuro-axonal injury [11]. While the authors postulated that this effect was mediated by remyelination induced by estrogen receptor beta ligand treatment, as previously shown in preclinical models, remyelination was not directly measured in the current trial. An ongoing trial is currently being conducted of estriol treatment for cognition in women with MS aged 18–55 (NCT01466114).

### Impact on Symptoms

Theoretically, pharmacologically reducing hormonal fluctuations using continuous OCs could in turn reduce fluctuations in menstrual and MS symptoms in MS, analogous to the strategy that has been used in epilepsy and migraine (9–11). Observational data are difficult to collect and thus far, a small prospective observational study identified only a small potential reduction in daily variability in symptoms [16].

In the aforementioned multicenter randomized controlled trial of OC combined with interferon beta, mood and fatigue scores were comparable across the groups over time at both time points. However, at month 24, the high-estrogen combined OC group showed worsening on the sexual function subscale of the 54-item MS quality-of-life questionnaire (*p* = 0.03) [16].

To summarize these findings, oral contraceptives do not appear associated with any adverse outcomes in MS. Some interventional data suggest that combined OCs may play a modest neuromodulatory role. While larger-focused studies are needed, continuous OCs might be considered when seeking to reduce symptomatic fluctuations, as has been done in other neurological conditions.

## Menopausal Hormone Therapy

Approximately two-thirds of all individuals diagnosed with MS will be females prior to the menopausal age, and therefore, this physiological transition is of epidemiological relevance in MS. Natural menopause is defined (retrospectively) as the final menstrual period (FMP) beyond which there are no menses for one year. Women with MS appear to approach this life transition at ages consistent with the general population, i.e., with a median age at natural FMP of 51 years [17–19]. Early menopause is defined as FMP occurring before age 45 years, and premature menopause is defined as FMP occurring before age 40 years.

**Fig. 1:**
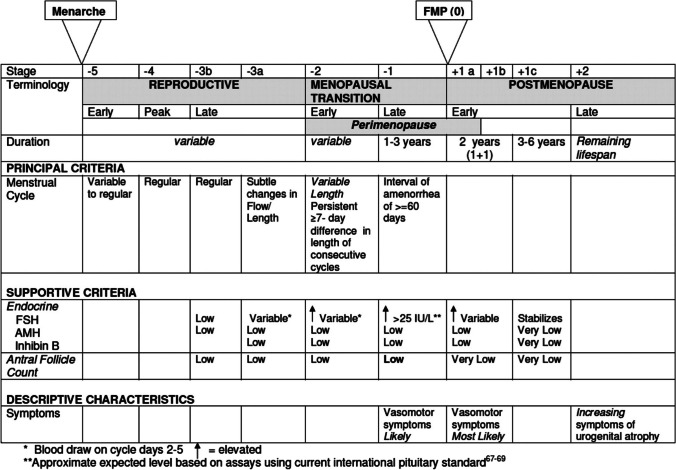
Stages of reproductive aging. Harlow SD, Gass M, Hall JE, Lobo R, Maki P, Rebar RW, et al. Executive summary of the Stages of Reproductive Aging Workshop + 10: addressing the unfinished agenda of staging reproductive aging. The Journal of clinical endocrinology and metabolism. 2012;97(4):1159–68. https://doi.org/10.1210/jc.2011-3362. [18]

While menopause is categorically defined based on the final menstrual period (Fig. 1), physiological reproductive aging occurs in more gradual, well-characterized phases [20]. Towards the end of the reproductive years, progesterone levels decline and estradiol levels fluctuate as menses become more irregular. The perimenopausal period encompasses these final cycling years through the first year following the final menstrual period. Vasomotor symptoms (hot flashes) are most common during this phase. The postmenopausal period—approximately one-third of total human lifespan—follows this perimenopausal period and is categorized as early (4 years) or late (thereafter).

According to the North American Menopause Society [21], menopausal hormone therapy is indicated both to treat menopausal vasomotor symptoms, to prevent bone density loss in the postmenopausal period, to treat hypoestrogenism (mood, cognition, above symptoms) caused by hypogonadism, surgical menopause, and premature ovarian failure, and to treat vulvovaginal symptoms. Systemic menopausal hormone therapy typically consists with an estrogen, such as estradiol and/or conjugated equine estrogens (Premarin), paired with a progestogen to protect the endometrial lining in women with preserved uterus. Local estrogen therapy can also be delivered intravaginally to provide relief against the genitourinary syndrome of menopause. Understanding of the risks and benefits of systemic menopausal hormone therapy has evolved substantially over the past 25 years. Many physicians currently practicing today trained since the results of the Women’s Health Initiative (WHI) raised problematic concerns about the safety of menopausal hormone therapy. A reinterpretation of these data and of subsequent data suggest that many of these risks do not apply to hormone therapy when given appropriately within the window of the menopausal transition. In fact, estrogen alone represents a significant but small elevated risk of thrombotic disease but otherwise risk reduction across a range of oncologic and cardiometabolic outcomes as well as all-cause mortality [21].

**Fig. 2:**
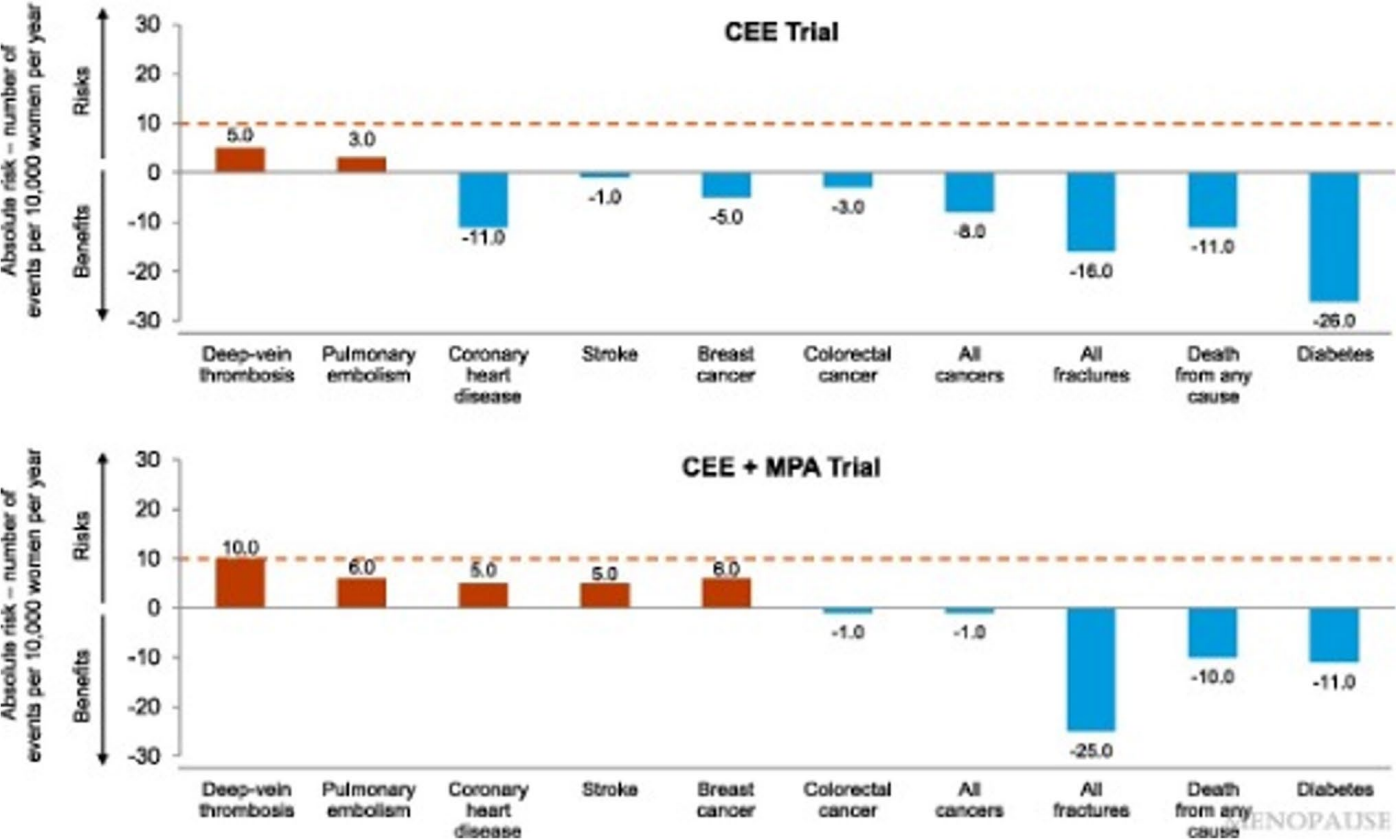
Overview of benefits and risks of systemic menopausal hormone therapy in women aged 50–59 years in the Women’s Health Initiative. The 2022 hormone therapy position statement of The North American Menopause Society. Menopause (New York, NY). 2022;29(7):767–94. https://doi.org/10.1097/gme.0000000000002028 [19]

With respect to menopause in women with MS, there are two distinct questions about the relevance of hormonal therapies. The first has to do with women’s experiences during the perimenopausal period, and the second has to do with the possible protective effects of hormonal therapy against neurodegeneration in the postmenopausal period (Fig. 2).

- a. Impact on Perimenopausal Symptoms
  During the perimenopausal period, women—regardless of their ambulatory function—can experience exacerbations across many symptoms—sleep, energy, mood, libido—that are due to either the underlying hormonal changes they are experiencing, or more directly due to the effect of vasomotor symptoms. This increase in symptomatic burden can arise rapidly, even in individuals with overall mild-moderate disease burden and good control of inflammatory activity, and out of proportion with any ambulatory dysfunction, confounding patients’ understanding of their overall MS status. In the clinic, common questions include “Is this my MS, my menopause, or both?” and, “Am I going crazy?”. For example, in an analysis of 59 female trial participants at midlife, mental well-being was significantly associated with sleep quality, depression, and hot flash interference, and not at all with ambulatory difficulty. [22] In addition, menopausal patients with MS also experience a number of other unmet needs including access to preventive care for healthy aging, mental health support, as well as evolving employment and family situations.
  Therefore, stabilizing vasomotor symptoms could play an important role in improving overall symptom control and quality of life in perimenopausal women with MS. While hormone therapy is considered an effective approach to VMS treatment [21], there are several non-hormonal approaches to hot flash control including antidepressants (e.g., selective norepinephrine reuptake inhibitors [23]) as well as the newly FDA-approved neurokinin-3 receptor antagonist Fezolinetant. [24–26].

### Interventional Studies

There are no observational studies evaluating the role of menopausal hormone therapy on MS or vasomotor symptoms in women with MS. Two small interventional studies assessed this specifically. The first was an 8-week Phase Ib/IIa trial randomizing 24 peri/postmenopausal women with MS and symptomatic hot flashes 1:1 to Premarin + the selective estrogen receptor modulator (SERM) bazedoxifene vs. placebo [27•]. Enrollment was protracted (34 months), partially due to concerns about hormone therapy safety. Participants randomized to the treatment group reported greater satisfaction and fewer missed doses. In terms of efficacy, Hot Flash Related Daily Interference scale at 2 months was lower in the treatment group vs. placebo group (median (IQR) of 4 (0.5, 14) vs. 9 (0, 33)), but differences were not significant. This trial underscored substantial hurdles to enrollment in hormone therapy trials due to prevailing concerns both by patients and their practitioners [27•].

In a second, open label baseline-controlled trial, 14 perimenopausal women with RRMS on stable moderate-efficacy or no DMT and 13 women without MS received 1 or 2 mg of estradiol and cyclical 10 mg dydrogesterone for one year [28]. At baseline, the MS group had more common depressive symptoms, but similar vasomotor and insomnia symptoms to the non-MS group. Over the 12-month study, there were no serious or unexpected adverse effects. VMS frequency decreased in both groups. Depressive symptoms decreased at 3 months (*p* = 0.031 with MS; *p* = 0.024 without MS) and the reduction was sustained at 12 months (*p* = 0.017; *p* = 0.042, respectively). Alleviation in insomnia symptoms was seen in participants without MS at 3 months (*p* = 0.029) and in those participants with MS suffering insomnia at baseline (*p* = 0.016 at 3 months; *p* = 0.047 at 12 months). Both groups improved their performance in PASAT, but no significant change was observed in SDMT, suggesting practice effects. MS activity at baseline was mainly stable, and no increase in activity was detected during MHT. While this trial was of significantly longer duration than the prior one, besides its small sample size, another major limitation of the trial was lack of placebo control in this population that has well-recognized placebo effects [29] as well as possible ‘phasing out’ of VMS over the course of the year.

Altogether then, one interpretation of the evidence to date in both the general and MS menopausal populations is that, beyond a specific risk of thromboembolism in more sedentary patients, there are likely no MS-specific reasons not to pursue hormonal therapy for treatment of VMS, and when hormone therapy is contraindicated, non-hormonal approaches should be used to alleviate symptomatic exacerbations and improve quality of life in this population.

- b. Impact in the Postmenopausal Period
  In the general population, features of menopause such as early and/or surgical menopause are associated with increased risk of cognitive decline and neuropathology. Less is known about trajectories of specific neurological diseases in the postmenopausal period. In MS, after the final menstrual period, a decrease in inflammatory attacks has been reported [30, Ladeira, 2018 #1659], as well as an acceleration in the slope of worsening of overall disability (EDSS) worsening reported in some [17, 30] but not all [31, 32] cohorts. Cohorts differed in numbers and in numbers of post-menopausal timepoint observations. More recently, acceleration in continuous, objective measures of neurodegeneration including the MS Functional Composite score, sNFL, and whole brain gray matter volume, was reported (Silverman, in prep).

### Effect on Inflammation

With respect to effects of menopause hormone therapy on inflammation, there are no interventional trials. Observational studies are limited, partly because of the very low (< 25%) rate of hormone therapy use in modern cohorts which both reduces statistical power and introduces marked treatment biases [17, 33, 34].

### Effect on Neurodegeneration

There are a number of putative benefits of menopausal hormone therapy in promoting resilience and preventing neurodegeneration. Mechanistically, E2-mediated neuroprotection has been reported to work primarily through estrogen signaling via two estrogen-binding receptor proteins ERα and ERβ. As individuals undergo menopause, the loss of circulating estrogen results in subsequent degradation of these receptors, and neuroprotective pathways that rely on estrogen are curtailed. Such pathways are many: E2 helps maintain the blood brain barrier, regulate apoptosis-related genes, provide response to neuronal damage through non-genomic signaling, and initiate antioxidant mechanisms [35]. In the general population, menopause, and its associated cessation of circulating estradiol (E2), has been correlated with cerebrovascular disease and dysfunction, sparking general interest in the use of menopausal hormone therapy to not only treat vasomotor symptoms of menopause but also to treat and prevent neurological disorders, such as dementia and Parkinsonism. Indeed, women who experience early menopause have been reported to face up to 5 times greater risk of mortality from neurological disorders than women not undergoing premature menopause [35]. However, the specific benefits of hormone therapy on cognitive and neurological function remain unclear. While observational studies suggested protective effects on cognition and dementia neuropathology [36, 37•, 38], the landmark interventional Women’s Health Initiative Memory Study confused the picture because of an overall increase in risk of dementia and mild cognitive impairment observed after estrogen therapy in women ages 65 or older [39], whereas further investigation into the Women’s Health Initiative Memory Study (WHIMS) via the WHIMS-Young study found that cognitive ability in women aged 50–55 who had taken part in the study were neither impacted positively nor negatively by estrogen therapy [40]. Similarly, another large scale controlled trial of women early in menopause, with a mean age of 53, found neither benefit nor harm from hormonal therapy on measures of memory and cognitive function, and found improvement in depression and anxiety symptoms in those randomized to treatment with oral conjugated estrogen (versus transdermal estradiol and placebo) [41]. Exogenous estradiol use was also shown to protect against global ischemia-induced neuronal cell death [42]. Outside of cognition, in the WHI estrogen-only trial, benefits in risk of chronic disease were noted for perimenopausal participants ages 50–59, with greater risk for late postmenopausal women [43]. In another randomized controlled trial, with its cohort split by early postmenopausal stage (less than 6 years after menopause) and late postmenopausal stage (greater than 10 years after menopause), a significant decrease in the rate of carotid artery intima-media thickness was noted only in the early postmenopausal group, with no effect observed in the late postmenopausal group [44]. This led to a hypothesized “window of opportunity” around menopause, during which hormone therapy may have protective benefits on cognition [45], though one large-scale trial found that estradiol had neutral impact to cognition regardless of postmenopausal timing [46].

In women with MS, an older observational study of menopausal hormone therapy and patient self-reported physical function in the Nurses Health Study [47•] reported better physical function in women who used systemic perimenopausal hormone therapy than women who did not, but these patients also reported better physical function at a pre-menopausal, pre-hormone therapy timepoint. Rather than demonstrating a protective effect of menopausal hormone therapy on neurodegeneration, this suggested a treatment bias—that women with less physical disability were more likely to receive age-appropriate standard of care which at the time of their menopause, included hormone therapy [47•]. More recent observational studies have not reported a protective effect of menopause hormone therapy on the observed worsening in function postmenopausally, with the notable bias that fewer than ¼ patients are treated [17, 33, 34] (Silverman, in prep). For example, a study of prospectively enrolled cases from the Danish MS registry identified 3325 women with RRMS, treated with a DMT, of whom 333 (10%) were ever on HT at some point during follow up. There was no association between hormone therapy, especially if used for < 5 years, and EDSS disability accrual [33].

To our knowledge, there are no interventional studies of menopausal hormone therapy for neuroprotection in postmenopausal women with MS.

To summarize these findings, menopausal hormone therapy is an effective treatment for menopausal symptoms and does not appear to be associated with elevated risk of adverse events overall, when used in the appropriate perimenopausal time window. It is, however, contraindicated in women with specific risk factors. To treat menopausal vasomotor symptoms when hormone therapy is contraindicated, non-hormonal agents, including antidepressants and the newly FDA-approved neurokinin-3 receptor antagonist fezolinetant, can be effective. More data are needed to inform whether hormonal therapy plays immunomodulatory or neuroprotective effects after menopause.

## Androgen Therapy

### Overview of Treatments

There are four androgen hormones: dihydrotestosterone (DHT), testosterone, androstenedione, and dehydroepiandrosterone (DHEA). Testosterone can be aromatized to estrogens and is the most concentrated androgen in male serum. DHEA binds to androgen receptors and ERα and ERβ and can be metabolized to testosterone and estrogens. Androstenedione can also be converted to estrogen, while DHT is the only androgen unable to do so and therefore works exclusively via testosterone receptors.

### Association Studies

Several early observational studies suggested that men with MS may experience hypogonadotropic hypogonadism, and/or have reported lower testosterone, DHEA, or DHEA-S levels in men and women with MS as compared to age-matched healthy controls [48–50].

#### Effect on Inflammation

##### Putative Effects

Testosterone has demonstrated anti-inflammatory properties and neuroprotective effects in EAE models and other animal models of autoimmunity [51, 52]. In EAE models, endogenous testosterone has been shown to be protective in some genetic backgrounds, whereas exogenous testosterone has benefits for all genetic backgrounds [52]. In several in vivo and in vitro studies, testosterone treatment has been shown to possibly have beneficial effects in Th1-mediated autoimmune diseases, showing evidence of a possible Th2-like shift and reduced production of inflammatory cytokines [52]. Further, T cell and B cell differentiation, as well as effector functions, may be affected by androgens [53].

##### Interventional Studies

A small, open-label, crossover phase II trial studied the effect of 12-month testosterone treatment (100 mg gel daily) in ten men with relapsing–remitting MS; each person was followed for a 6-month pre-treatment period, followed by 12 months on-drug. The trial suggested that this approach was safe and well-tolerated [54], but there was no significant effect on gadolinium-enhancing lesion quantity or volume, indicating a neutral impact to inflammation and white matter lesion burden.

#### Effect on Neuroprotection and Repair

In the aforementioned trial, while testosterone treatment did not seem to influence disease activity, it was reportedly associated with improvement in cognitive performance (*p* < 0.001) [54], as well as with a significant voxel-wise gray matter increase (p ≤ 0.05 corrected) in the treatment period compared to decrease in the earlier 6 month pre-treatment period (p ≤ 0.05) [55]. Overall the small sample size, open label design, heterogeneity in MS DMT use and surprising effect of testosterone treatment on brain volume increase, suggest that larger, controlled, trials are needed to evaluate its effect on MS inflammation, progression, and/or symptoms.

To summarize these findings, to date, testosterone therapy can be considered in individuals with MS with clinical hypogonadism and according to current clinical guidance of the general population linked to the Endocrine Society [56]. Caution is advised in individuals at higher risk of thrombotic events. More data are needed before determining whether androgen therapy has any clear immunomodulatory or protective effect in clinical MS populations.

## Selective Estrogen and Androgen Receptor Modulators (SERM and SARM)

Selective estrogen receptor modulators (SERMs) are increasingly used in the settings of breast cancer, osteoporosis, and postmenopausal symptoms [57], whereas and selective androgen receptor modulators (SARMs) have positive clinical potential for treatment of cancer-related cachexia, benign prostatic hyperplasia, hypogonadism, and breast cancer [58]. SERMs should theoretically avoid certain risks of estradiol, such as negative carcinogenic or cardiovascular effects, due to their more selective tissue targeting.

### SERMs: Putative Effects on Myelin Repair

SERMs, selective nuclear estrogen receptor agonists or antagonists, can induce both positive and negative estrogenic impacts on the central nervous system, with evidence of neuroprotective and cognitive impact in both animal and human models [59]. SERMs have putative effects on myelin repair. Mechanistically, only newly differentiated oligodendrocytes, not existing oligodendrocytes, are able to participate in myelin repair but in MS, OPC differentiation is blocked—potentially due to accumulated myelin proteins [60•, 61]—and bypassing this block is critical to promote myelin repair. In a high-throughput screen, certain SERMs (lasofoxifene, bazedoxifene, and tamoxifen, but not estradiol) were found to promote OPC differentiation and thereby have remyelination potential that was confirmed in vivo [60•]. Interestingly, nuclear ERs do not appear necessary for SERMs to promote myelin repair; EBP (encoding 3β-hydroxysteroid-Δ8,Δ7-isomerase), an enzyme in the cholesterol biosynthesis pathway, represents a potential target for this effect [60•, 62–64].

Among these SERMs, tamoxifen, commonly used for adjuvant therapy for early breast cancer treatment, is able to cross the blood–brain barrier, and with its well-documented safety profile, has been of interest for clinical use in remyelination. However, studies on cognition and tamoxifen in the non-MS population have shown inconsistent results; this heterogeneity may be partially attributed to differing age groups, bucketing of hormonal therapies, and uses of non-breast cancer controls vs. breast cancer controls [65•]. Some studies indicate associations with tamoxifen and impaired verbal learning and memory [66] and higher risk of Alzheimer’s disease [67], while others, including a large retrospective cohort study of 57,843 women aged 45 to 90 with breast cancer, reported lower risk of Alzheimer’s disease and dementia after tamoxifen use [68]. Yet, others show no association between tamoxifen and cognition [65•].

In terms of preclinical evidence for remyelinating effects of SERMs, demyelinated rats treated with tamoxifen have shown in vivo accelerated remyelination, and in vitro, tamoxifen had potent effects on oligodendrocyte precursor cell (OPC) [61]. However, tamoxifen treatment is limited to a duration of 5 years due to its high risk: benefit ratio [59]. BZA, compared with other high-efficacy SERMs, has been well-tolerated in clinical trials with a well-documented safety profile [69]. It is currently approved by the European Medicines Agency (EMA) for postmenopausal osteoporosis and is FDA-approved in combination with conjugated estrogen (Premarin) for menopausal vasomotor symptoms and postmenopausal osteoporosis. Using an in vivo murine model of demyelination (lysolecithin-induced focal demyelinating lesion in the corpus callosum), BZA was found to strongly enhance OPC differentiation and remyelination [60•]. In addition, human embryonic stem cell derived OPCs were found to significantly differentiate into mature oligodendrocytes and with treatment of BZA [57]. A double blind, randomized, controlled Phase II clinical trial (NCT04002934) is currently investigating remyelinating effects of BZA in postmenopausal women with MS [70]. Currently, there is no clinical data on SERM use for remyelination, and no therapy approved for remyelination, underscoring the need for further research on clinical application of SERMs in MS.

### SARMs

**Fig. 3:**
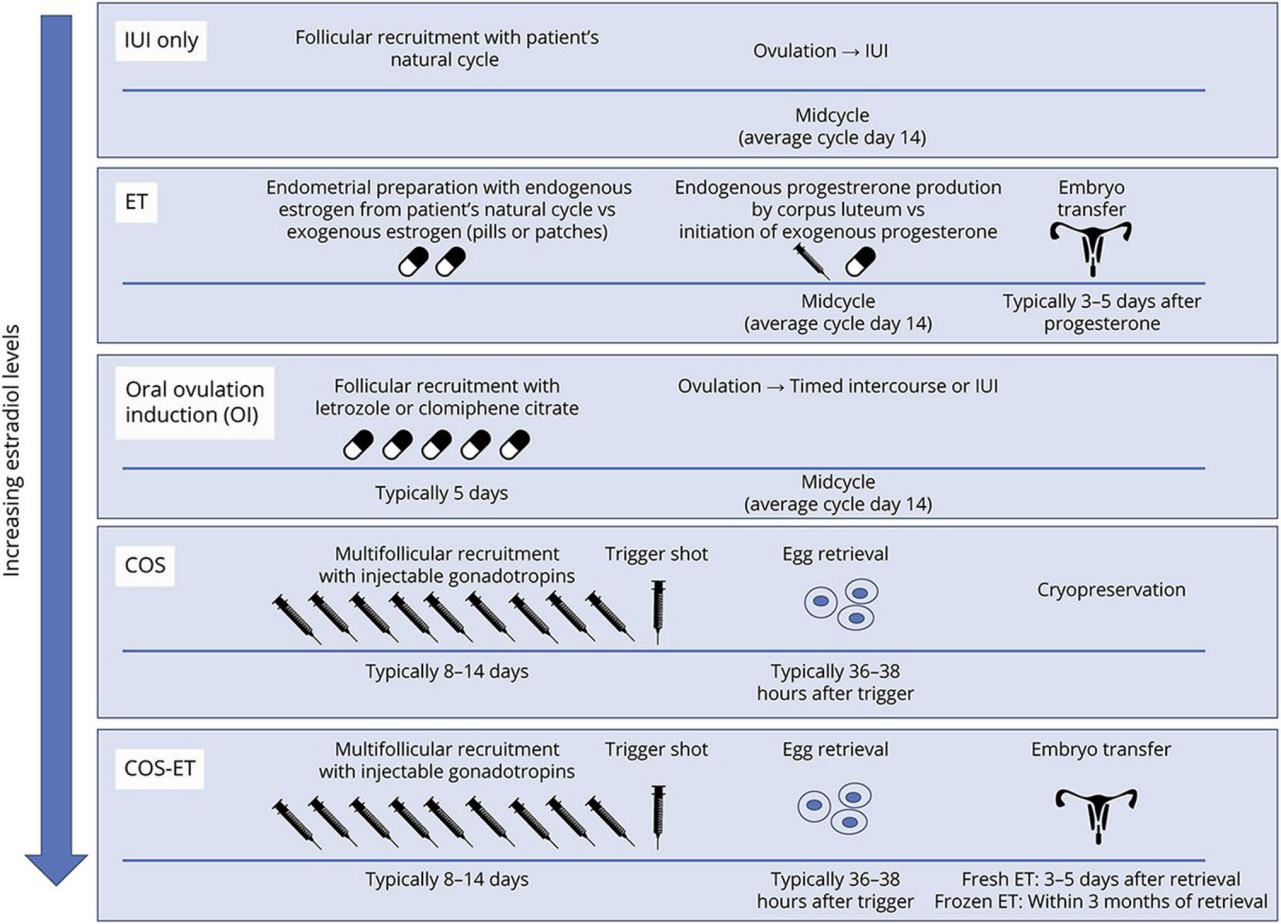
Overview of common fertility treatments, organized based on hypothetical risk of MS inflammatory activity. Graham EL, Bakkensen JB, Anderson A, Lancki N, Davidson A, Perez Giraldo G, et al. Inflammatory Activity After Diverse Fertility Treatments: A Multicenter Analysis in the Modern Multiple Sclerosis Treatment Era. Neurology(R) neuroimmunology & neuroinflammation. 2023;10(3). https://doi.org/10.1212/nxi.0000000000200106. [71]

Similar to SERMs, the tissue-selective nature of SARMs makes it an appealing candidate for safer androgen-based therapy without as many of its associated side effects. However, little data, and no clinical data, on SARM efficacy in remyelination and oligodendrocyte differentiation exist. One preclinical study found a potential positive effect on neurodegenerative disease: in murine models, the novel SARM NEP28 was found to increase the activity of the enzyme neprilysin, a known amyloid beta-degrading enzyme, indicating a potential use for Alzheimer’s disease [71]. As these products are increasingly used as performance enhancing agents and are also currently being studied clinically with positive benefits for use in breast cancer, cancer-related cachexia, benign prostatic hyperplasia, and hypogonadism [58], it will be possible to evaluate their effect in individuals with MS (Fig. 3).

## Fertility Treatments

An increasing number of individuals are undergoing diverse fertility treatments for a range of indications. This can include controlled ovarian stimulation for egg retrieval for fertility preservation, embryo transfer from donor eggs/embryos, and intrauterine insemination from donor sperm, as well as the ‘spectrum’ of controlled ovarian stimulation, egg retrieval, and embryo transfer in “classic” in vitro fertilization. Each of these situations requires a different dosing and formulation of exogenous hormones [72•].

Historically, five small trials reported an elevated risk of relapses after fertility treatment (FT), especially in unsuccessful cycles and those in which a gonadotropin hormone-releasing hormone (GnRH) agonist was used [73]. However, 4 more recent studies of women in the therapeutic disease-modifying therapies “DMT” era including not only in vitro fertilization (IVF) but also other fertility treatment protocols have not replicated these data [72•, 74, 73]. For example, Graham et al. analyzed 124 fertility treatment cycles and reported no observed elevated relapse risk after each cycle. Further, relapse rates did not vary by controlled ovarian stimulation protocol [72•].

Altogether then, the current data suggest that for women who are actively managed using current standards of DMT treatment surrounding their fertility treatments, there is no elevated risk of relapses and no MS-related indication to forego fertility treatments should these be otherwise indicated. To optimize inflammatory as well as quality of life and emotional outcomes, care coordination between neurological and fertility experts is advised, with careful DMT planning and emotional support provided as needed.

## Gender Affirming Hormone Therapy

There is increasing recognition of the needs by sexual and gender minorities for specialized neurological care [75, 76]—including in MS [77–80]. These populations face a number of concerns including limited cultural and clinical competence from neurologists [75] and health systems, minority stress, and intersection of minority group identity and neurological disability.

Among transgender individuals, gender affirming hormones could pose specific concerns. Such hormones include, for feminizing therapies, estrogens (primarily 17-beta-estradiol given orally, transdermal, sublingual, or intramuscular), suppression of testosterone production (high-dose spironolactone; 5-alpha reductase inhibitors such as finasteride and dutasteride which block conversion of testosterone to more potent dihydrotestosterone; gonadotropin-releasing hormone agonists, and occasionally, progestogens). For masculinizing therapies, various formulations of topical or injectable testosterone are used, and occasionally progestogens [26].

A retrospective national record-linkage study from British National Health Service data compared 1157 males and 2390 females with “gender identity disorders” with 4.6 million male and 3.4 million female controls with mostly minor conditions [81]. They reported an elevated adjusted relative risk of MS following a diagnosis of “gender identity disorder” in males (i.e., trans-women) of 6.63 (95% CI = 1.81–17.01, significant at *p* = 0.0002), relative to the reference male cohort. This was based on just 4 observed cases and 0.6 expected. There was no increased risk of MS in trans-men compared with the reference female cohort. While specific information was not available regarding treatments used, the data implied that gender affirming treatments, specifically reduction in androgen levels or exogenous estrogens, could increase the risk of MS in biological males. Similar observations have been made for other autoimmune conditions, such as systemic lupus erythematosus [82–86]. Further, an elevated risk of cerebrovascular disease, possibly due to coagulopathic effects, has been reported with gender-affirming estrogen use [87, 88] and possibly also progestogens [89], which could also increase neurological vulnerability of trans-women receiving exogenous hormones.

To summarize these findings, gender-affirming estrogenic therapies could be associated with some increase in risk of inflammatory or thrombotic sequelae. However, given the limitations of the studies to date, including low numbers, lack of details about exogenous hormone exposures and ascertainment biases, prospective, detailed record linkage studies are needed in larger health system datasets to understand the true risks and consequences for gender-diverse individuals with MS.

## Conclusion

The potential for exogenous hormones to influence the risk and course of MS remains of high interest in the field. There is increasing attention to these hormones—used in doses and for indications as diverse as fertility treatments, contraception, gender affirmation, androgen therapy, adjuvant oncologic treatment, or menopausal vasomotor symptoms—and their effects on MS risk, inflammatory activity, or neuroprotection. Due to many confounds, large randomized clinical trials are required to determine a true effect. To date, this has only been accomplished for combined oral contraceptives [8] and estriol [11]—both showing a modest effect on inflammatory activity when given as an add-on to first-line self-injectable DMTs, and the latter showing also potential neuroprotective effects. For the other indications, newer data in larger actively treated cohorts have not confirmed earlier concerns about fertility treatments and relapse activity, instead showing no elevated risk of relapses after diverse fertility treatments. However, timing of DMT use and fertility treatments should be coordinated. Androgens have shown anti-inflammatory properties and neuroprotective effects in EAE models, but to date, only a small uncontrolled interventional study has been performed in men with MS [54]. Selective estrogen receptor modulators (SERMs) and selective androgen receptor modulators (SARMs) are increasingly used for a range of indications—these may theoretically avoid the negative effects of estrogen and androgens due to their more selective tissue targeting. One potential attractive mechanistic target of SERMs concerns promotion of OPC differentiation and thus remyelination potential [60•], including one currently in a controlled clinical trial [70]. Little data on SARMs exist in MS, with one murine model reporting a potential positive effect on neurodegenerative disease with the novel SARM NEP28 [71]. For gender affirming treatments, some data suggest a possible elevated risk of inflammation in trans-women, presumably as a result of estrogens.

For the 80–90% of females with MS who will develop MS prior to the menopausal transition, more research is needed to determine whether treatment of vasomotor symptoms substantially influences overall well-being and quality of life during this transition. Two small interventional trials on women with MS both found difficulty in enrollment due to patient and practitioner concern over potential hormonal therapy risks [27•, 28]. However, they reported qualitative satisfaction, lower hot flash disturbance, and reductions in vasomotor symptoms, depressive symptoms, and insomnia, indicating that hormonal treatment for menopause symptoms should not be ruled out because of an MS diagnosis. Finally, the possible neuroprotective effects of estrogens in postmenopausal women, as well as of androgen treatments in postmenopausal men, require ongoing investigation. Interpretation of menopausal hormone therapy effects has evolved significantly over the past 25 years, and, when administered over an appropriate window within the menopausal transition, it appears largely safe, with the exception of elevated risk of thromboembolism in more sedentary patients. Its effect on neuroprotection remains to be determined, and longitudinal trials are needed in this population.
